# Virtual Monoenergetic Imaging of Lower Extremities Using Dual-Energy CT Angiography in Patients with Diabetes Mellitus

**DOI:** 10.3390/diagnostics13101790

**Published:** 2023-05-18

**Authors:** Giuseppe Mauro Bucolo, Tommaso D’Angelo, Ibrahim Yel, Vitali Koch, Leon D. Gruenewald, Ahmed E. Othman, Leona Soraja Alizadeh, Daniel P. Overhoff, Stephan Waldeck, Simon S. Martin, Silvio Mazziotti, Giorgio Ascenti, Alfredo Blandino, Thomas J. Vogl, Christian Booz

**Affiliations:** 1 Division of Experimental Imaging, Department of Diagnostic and Interventional Radiology, University Hospital Frankfurt, 60596 Frankfurt am Main, Germany; 2 Department of Biomedical Sciences and Morphological and Functional Imaging, University of Messina, 98122 Messina, Italy; 3 Department of Radiology and Nuclear Medicine, Erasmus MC, 3015 GD Rotterdam, The Netherlands; 4 Department of Diagnostic and Interventional Radiology, University Hospital Frankfurt, 60596 Frankfurt am Main, Germany; 5 Department of Neuroradiology, University Hospital Mainz, 55131 Mainz, Germany; 6 Department of Diagnostic and Interventional Radiology and Neuroradiology, Bundeswehr Central Hospital Koblenz, 56072 Koblenz, Germany; 7 Department of Radiology and Nuclear Medicine, University Medical Centre Mannheim, Medical Faculty Mannheim, Heidelberg University, 68167 Mannheim, Germany; 8 Institute of Neuroradiology, University Medical Centre, Johannes Gutenberg University Mainz, 55099 Mainz, Germany

**Keywords:** virtual monoenergetic imaging, dual energy, CT angiography, lower extremities

## Abstract

Background: Type 2 diabetes mellitus (DM) is the most common metabolic disorder in the world and an important risk factor for peripheral arterial disease (PAD). CT angiography represents the method of choice for the diagnosis, pre-operative planning, and follow-up of vascular disease. Low-energy dual-energy CT (DECT) virtual mono-energetic imaging (VMI) has been shown to improve image contrast, iodine signal, and may also lead to a reduction in contrast medium dose. In recent years, VMI has been improved with the use of a new algorithm called VMI+, able to obtain the best image contrast with the least possible image noise in low-keV reconstructions. Purpose: To evaluate the impact of VMI+ DECT reconstructions on quantitative and qualitative image quality in the evaluation of the lower extremity runoff. Materials and Methods: We evaluated DECT angiography of lower extremities in patients suffering from diabetes who had undergone clinically indicated DECT examinations between January 2018 and January 2023. Images were reconstructed with standard linear blending (F_0.5) and low VMI+ series were generated from 40 to 100 keV, in an interval of 15 keV. Vascular attenuation, image noise, signal-to-noise ratio (SNR), and contrast-to-noise ratio (CNR) were calculated for objective analysis. Subjective analysis was performed using five-point scales to evaluate image quality, image noise, and diagnostic assessability of vessel contrast. Results: Our final study cohort consisted of 77 patients (41 males). Attenuation values, CNR, and SNR were higher in 40-keV VMI+ reconstructions compared to the remaining VMI+ and standard F_0.5 series (HU: 1180.41 ± 45.09; SNR: 29.91 ± 0.99; CNR: 28.60 ± 1.03 vs. HU 251.32 ± 7.13; SNR: 13.22 ± 0.44; CNR: 10.57 ± 0.39 in standard F_0.5 series) (*p* < 0.0001). Subjective image rating was significantly higher in 55-keV VMI+ images compared to the other VMI+ and standard F_0.5 series in terms of image quality (mean score: 4.77), image noise (mean score: 4.39), and assessability of vessel contrast (mean value: 4.57) (*p* < 0.001). Conclusions: DECT 40-keV and 55-keV VMI+ showed the highest objective and subjective parameters of image quality, respectively. These specific energy levels for VMI+ reconstructions could be recommended in clinical practice, providing high-quality images with greater diagnostic suitability for the evaluation of lower extremity runoff, and potentially needing a lower amount of contrast medium, which is particularly advantageous for diabetic patients.

## 1. Introduction

Type 2 diabetes mellitus (DM) is the most common metabolic disorder in the world, with an incidence and prevalence greater than 80% in patients living in low-to-middle-income countries [1]. Metabolic dysregulation causes high blood glucose levels, which damage the heart, blood vessels, kidneys, eyes, and nerves through different pathogenetic mechanisms [1]. Because of its high prevalence, DM is one of the major risk factors for cardiovascular disease [2]. 

Peripheral arterial disease (PAD) represents a major DM complication, increasing the risk of ischemic ulceration, gangrene, amputation, and death [3,4].

Moreover, metabolic alterations caused by DM lead to fibrosis and glomerular sclerosis, resulting in chronic kidney disease (CKD), affecting about 40% of diabetic patients [5]. Renal dysfunction gradually progresses into different stages, from a slight functional decline to severe kidney insufficiency. The risk of obliterating arterial disease of the lower extremities is higher in patients with severe renal insufficiency than in the general population [6,7].

CT angiography (CTA) represents the method of choice for the diagnosis, pre-operative planning, and follow-up of vascular disease [8]. In fact, it has practically replaced digital subtraction angiography, which currently plays almost an exclusively interventional role.

Nowadays, dual-energy CT (DECT) technology is increasingly available and possesses several advantages in clinical use due to its different applications such as virtual mono-energetic imaging (VMI), which represents a beneficial innovation with several ranges of use, particularly in cardiovascular imaging [9].

Through the post-processing of raw data obtained from high- and low-energy radiation beams, low-keV VMI images (40–75 keV) close to the iodine K-edge (33.17 keV) improve image contrast and iodine signal. In this context, studies have demonstrated that the VMI+ reconstruction algorithm provides the best image contrast with the least possible image noise in low-keV reconstructions, potentially leading to a reduction in contrast medium dose [10,11,12,13]. 

Patients suffering from severe PAD, particularly in cases of significant stenosis or occlusion of main upstream vessels, show reduced iodine signal in their lower extremity vessels.

However, to the best of our knowledge, no studies have been published evaluating low-energy VMI+ reconstructions of lower extremity CTA in patients with DM, who have a high risk of suffering from severe PAD. 

Thus, the purpose of our study is to evaluate the use of VMI+ DECT reconstructions in order to identify the optimal energy level for the evaluation of the vascular structures of the lower extremities, which, in turn, would improve image quality and assessment.

## 2. Material and Methods

This retrospective study was approved by our institutional review board and informed consent was obtained.

### 2.1. Study Population

We reviewed our institutional databases to identify patients suffering from diabetes who had undergone DECT angiography scans of their lower extremities between January 2018 and January 2023. Patients with metal artifacts (*n* = 4) or moving artifacts (*n* = 7) affecting the proximal anterior tibial artery (ATA), fibular artery (FA), or posterior tibial artery (PTA) were excluded. The final study population was 77 patients with ages between 58 years and 92 years. 

**Figure 1:**
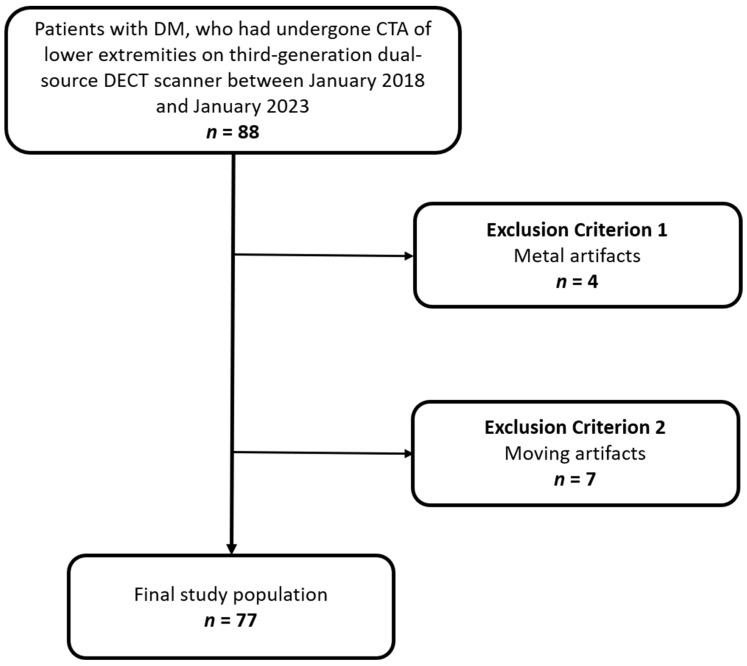
Flowchart showing patient inclusion and exclusion criteria for this study. DM: diabetes mellitus; CTA: CT angiography; DECT: dual energy CT.

Figure 1 shows the patient selection flowchart. 

### 2.2. DECT Scan Protocol

All scans were performed on a third-generation dual-source DECT scanner (SOMATOM Force, Siemens Healthineers, Forchheim, Germany). Patients were examined in supine position and cranio-caudal direction. Preliminary, anteroposterior, and lateral scout scans were obtained from the lower abdomen to the forefoot. 

The study protocol consisted of unenhanced, angiographic, and venous phases, the latter two acquired in DE mode. 

Contrast medium (Iomeron 400 mgI/mL, Bracco, Milan, Italy) was intravenously injected through a superficial vein of the forearm in order to guarantee an iodine delivery rate of 1.6 mgl/s at a flow rate of 4 mL/s, followed by a bolus of 40 mL of saline. 

A bolus tracking region of interest (ROI) was positioned on the infrarenal abdominal descending aorta. The angiographic phase started 7 s after reaching the 120 HU trigger threshold. The venous phase was performed 70 s after contrast injection.

The scans were conducted from the distal abdominal aorta down to the forefoot.

Acquisition parameters used for native single-energy scans were: tube, 70 kV and 170 mAs per rotation; rotation time, 0.5 s; pitch, 0.6; and collimation, 2 × 192 × 0.6 mm. For dual-source DECT imaging scan parameters were the following: tube A, 90 kV and 73 mAs per rotation; tube B, Sn 150 kV with tin filter and 45 mAs per rotation; rotation time, 0.5 s; pitch, 0.6; and collimation, 2 × 192 × 0.6 mm.

### 2.3. Image Reconstructions

All images with a section thickness of 1.0 mm and an increment of 1.0 mm were reconstructed using a soft tissue convolution kernel (Qr40; Siemens). Conventional images were reconstructed using linear blending with a blending factor F_0.5 (50% 90 kV, 50% 150 kV spectra) equivalent to single-energy polychromatic 120 kV acquisition.

DECT data were transferred to a dedicated postprocessing workstation (syngo.via version VA30, Siemens Healthineers, Forchheim, Germany), and VMI series were reconstructed at energy levels ranging from 40 to 100 keV in 15-keV increments using the dedicated dual-energy software (CT Dual Energy, Siemens Healthineers).

### 2.4. Objective Image Evaluation

One radiologist with 5 years of experience in CTA quantified the attenuation values [HU] and standard deviation (SD) in reconstructed series in the ATA, fibular artery FA, and PTA.

A total of 10 ROIs with sizes ranging from 0.01 to 0.22 cm^2^ were drawn within the proximal segment of the vessel (five per side), covering the largest possible lumen and avoiding vessel borders and calcified and non-calcified plaque equally for each reconstruction. Average values of the measurements were used for statistical analyses.

Completed occluded vessels or those with severe stenosis were excluded from the objective analysis. 

Attenuation values and *SD* measurements of subcutaneous fat and iliopsoas muscle were recorded for the evaluation of signal-to-noise ratio (*SNR*) and contrast-to-noise ratio (*CNR*), calculated using the following formulas:

$$SNR=\frac{HU_{artery}}{SD_{fat}};\;CNR=\frac{\left(HU_{artery}-HU_{muscle}\right)}{SD_{fat}}.$$

The *SD* of subcutaneous fat was used as the reference of image noise.

### 2.5. Qualitative Image Analysis 

Three board-certified radiologists (*BLINDED* with 6–10 years of experience in vascular imaging) evaluated VMI+ reconstructions from 40 keV to 100 keV and the standard F_0.5 series. Image evaluation was performed with a conventional picture archiving and communication system workstation (Centricity, version 4.2; GE Healthcare, Solingen, Germany). The selected criteria followed a five-point scale to assess the overall image quality (1 = very poor, 5 = optimal), the image noise (1 = major noise, 5 = none), and the diagnostic assessability of vessel contrast (1 = non diagnostic, 5 = optimal) (Table 1). Each reader was free to adjust window settings and scroll through the whole stack of CT series. 

### 2.6. Statistical Analysis 

Statistical analysis was performed using statistical software (MedCalc version 12.7.2; MedCalc Software, Ostend, Belgium; Excel 365, Microsoft, Redmond, WA, USA).

Data distribution was evaluated using the Kolmogorov–Smirnov test. The comparisons of values between each VMI reconstruction were assessed using repeatedly measured analysis of variance tests for normal distribution data and the Friedman test for non-normal data. 

The statistically significant difference was indicated by a *p* value less than 0.05. 

All continuous variables are shown as mean and standard deviation (SD). 

## 3. Results

### 3.1. Population Characteristics

Our final study cohort consisted of 77 patients (mean age: 79.17 ± 6.46; range: 58–92), including 41 males (mean age: 78.93 ± 6.05; range: 58–90 years) and 36 females (mean age: 79.44 ± 6.99; range: 64–92). No preselection based on BMI, age, sex, or other characteristics was performed.

Patients also presented the following diagnosed comorbidities: peripheral arterial disease (34; 44%), hypertension (43; 56%), chronic kidney disease (CKD) (16; 20%), coronary artery disease (23; 30%), and previous myocardial infarction (9; 12%). Table 2 shows all patient characteristics. 

Among a total of 231 arterial segments examined, we excluded a total of 17 arteries (ATA: 8; FA: 4, and PTA: 5) because of total occlusion (*n* = 13) or severe stenosis (*n* = 4) that did not allow drawing an indicative ROI. 

### 3.2. Quantitative Assessment

**Figure 2:**
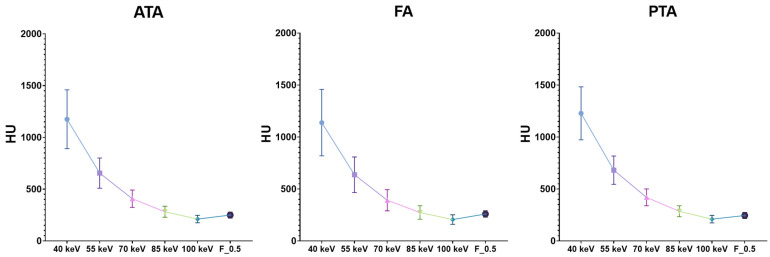
The graphs show the attenuation values (HU) distributions of VMI+ algorithms at various keV levels (from 40 keV to 100 keV) and the standard F_0.5 image series grouped by vessels. The highest attenuation values are present in 40-keV VMI, with the mean of all vessels being 1180.41 ± 45.09 HU. ATA: anterior tibial artery; FA: fibular artery; PTA: posterior tibial artery.

Attenuation values were highest in the 40 keV VMI+ series compared to the other VMI reconstructions (*p* < 0.0001), with all vessels’ average value being 1180.41 ± 45.09 HU, while standard linearly blended F_0.5 reconstructions had a value of 251.32 ± 7.13 HU. Subsequently, image noise in the 40-keV VMI+ series was 40.34 ± 5.57, the highest compared to other reconstructions (Figure 2).

Moreover, calculated SNR and CNR were significantly higher in the 40-keV VMI+ series compared to the other VMI+ reconstructions and the standard linearly blended F_0.5 reconstruction (*p* < 0.0001), with all vessels’ average values being 29.91 ± 0.99 and 28.60 ± 1.03, respectively. All the quantitative measurements are displayed in Table 3. 

**Figure 3:**
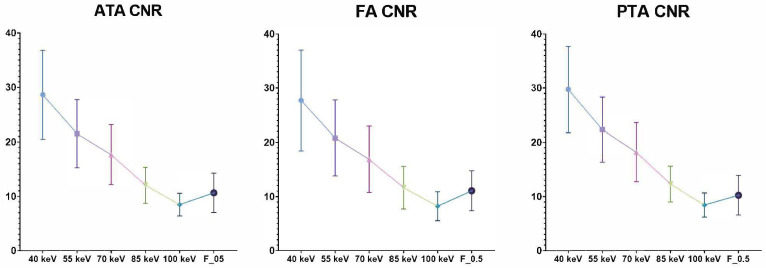
CNR distributions of VMI+ algorithms at various keV levels (from 40 keV to 100 keV) and the standard F_0.5 image series grouped by vessels. The graphs show higher CNR in low-keV reconstruction, especially in 40-keV VMI+ reconstructions (mean all vessels CNR: 28.60 ± 1.03 at 40-kev VMI+). CNR: contrast-to-noise ratio; ATA: anterior tibial artery; FA: fibular artery; PTA: posterior tibial artery.

**Figure 4:**
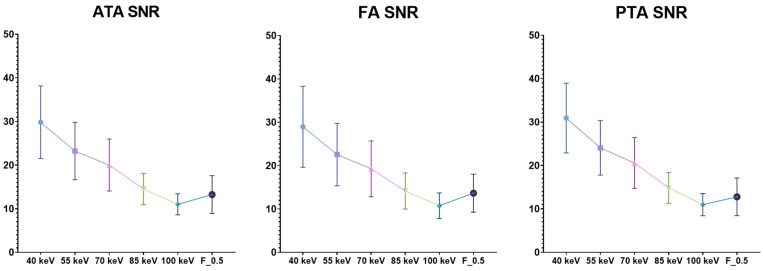
SNR distributions of VMI+ algorithms at various keV levels (from 40 keV to 100 keV) and the standard F_0.5 image series grouped by vessels. The graphs show higher SNR in low-keV reconstruction, especially in 40-keV VMI+ reconstructions (mean all vessels SNR: 29.91 ± 0.99 at 40-keV VMI+). SNR: signal-to-noise ratio; ATA: anterior tibial artery; FA: fibular artery; PTA: posterior tibial artery.

SNR and CNR have shown a progressive and significant reduction with energy levels increasing in the studied VMI+ reconstructions (Figure 3 and Figure 4).

### 3.3. Qualitative Image Analysis

Subjective image rating demonstrated the best image quality in the 55-keV VMI+ series (mean score, 4.77) followed by the 70-keV VMI+ series (mean score, 4.34), 40 keV-VMI+ (mean score, 4.22) and F_0.5 (mean score, 3.98) (*p* < 0.001). Inter-reader agreement was excellent for all VMI+ series as well as for F_0.5 (κ > 0.83; 95% CI, 0.76–0.90).

Image noise was rated with mean scores of 4.39 (55-keV VMI+ series), 4.30 (70-keV VMI+ series), 4.22 (85-keV VMI+ series), 4.07 (F_0.5), and 3.82 (40-keV VMI+ series) (*p* < 0.001). Inter-reader agreement was excellent for all VMI+ series as well as for F_0.5 (κ > 0.80; 95% CI, 0.72–0.87).

The diagnostic assessability of vessel contrast was rated best in 55-keV VMI+ series (mean score, 4.57), followed by 70-keV VMI+ series (mean score, 4.23), 40-keV VMI+ (mean score, 4.02), and F_0.5 (mean score, 3.78) (*p* < 0.001). Inter-reader agreement was excellent for all VMI+ series as well as for F_0.5 (κ > 0.82; 95% CI, 0.75–0.88).

## 4. Discussion

Our results show contrast and image quality improvement in low-keV (40 to 70-keV) VMI+ DECT reconstructions for the evaluation of lower extremities in patients with DM in comparison to other VMI+ energy levels and conventional image series (F_0.5). 

**Figure 5:**
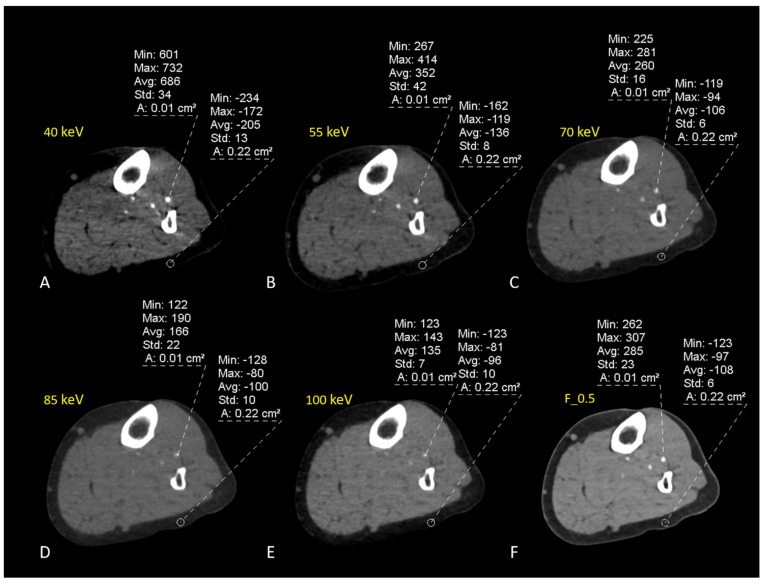
DECT-angiography scan of the left leg of a 65-year old woman suffering from DM and foot pain. Axial-VMI+ reconstructions ((**A**–**E**); from 40 keV to 100 keV) were created with dedicated post-processing software. The ROIs were drawn within the anterior tibial artery and subcutaneous fat on each VMI and in standard linearly blended F_0.5 reconstructions (**F**). Mean attenuation values increase at low keV-VMI. Noise is calculated as the standard deviation of subcutaneous fat.

Overall, average attenuation values increased in low-keV VMI+ series (40-55 keV) by about 60% compared to conventional image series (F_0.5), with a noise increment of about 40%. The VMI+ algorithm provides a mild increment of noise without affecting the images and offers high quality and diagnostic value at low energy levels (Figure 5). 

Objective evaluation of image quality showed an increase in CNR and SNR as the energy level decreased. Our results demonstrated the best CNR and SNR in the 40-keV VMI+ series, with results more than two times higher than standard F_0.5 reconstructions.

**Figure 6:**
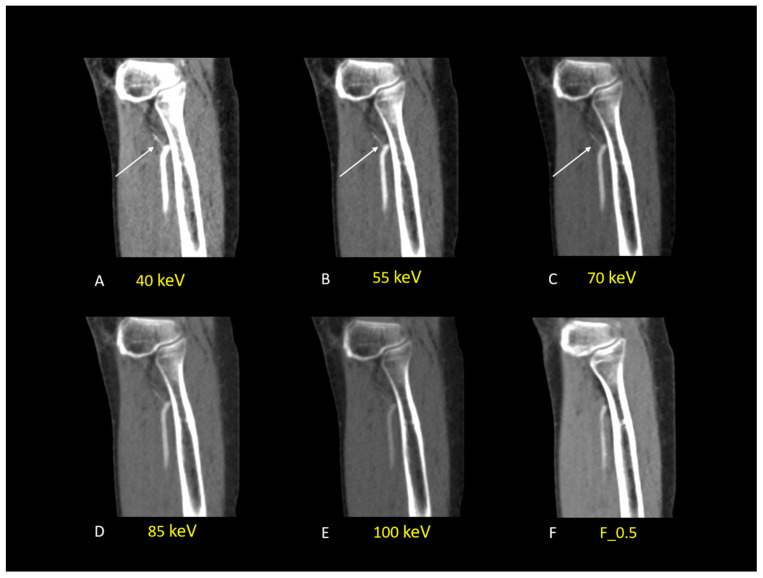
DECT-angiography scan of left leg in a 72-year-old man with DM and known PAD. Sagittal VMI+ reconstructions ((**A**–**E**); from 40 keV to 100 keV) are displayed. Low keV-VMI reconstructions show the ATA with high contrast quality and provide a good representation of a secondary branch (white arrow), which is missed at 85-100 keV and in the standard linearly blended F_0.5 image (**F**).

In addition, low-keV VMI+ series (40 to 70-keV) received a superior rating compared to other VMI+ energy levels and the standard F_0.5 series regarding the suitability of images for the subjective evaluation of limb arteries, enabling the identification of secondary arterial branches (Figure 6).

Compared to objective analyses, the subjective evaluation demonstrated superior ratings for the image quality, image noise, and assessability of vessel contrast in 55-keV VMI+ reconstructions compared with 40-keV, most probably due to the visual impression of high noise levels at 40-keV.

Nowadays, DECT is increasingly available and possesses several advantages due to its different applications in clinical use. Through the post-processing of raw data obtained from high- and low-energy radiation beams, different VMI datasets can be reconstructed into a specific monoenergetic level [14]. 

Low energy VMI provides an increase in image contrast; in particular, low-keV levels (40–75 keV) near the iodine K-edge (33.17 keV) are increasingly used for hypervascular lesions and vessel evaluation, enabling a high vascular contrast [15].

Initially, a standard VMI algorithm was severely impaired by high image noise at low energy levels, precluding the possibility of using energy levels such as 40–50 keV [15,16]. Thus, a new reconstruction algorithm called VMI+ was developed to obtain the best image contrast with the least possible image noise in low-keV reconstructions, using an additional frequency-split analysis instead of the conventional VMI algorithm [10].

Therefore, VMI+ reconstructions provide numerous advantages not only in the cardiovascular field, but also in abdominal evaluation, improving image quality, lesion detection, and diagnostic accuracy [17,18,19,20,21]. 

Similarly, other DECT applications, such as iodine maps and virtual non-contrast (VNC) imaging, demonstrated lots of benefits in clinical use in the field of cardiovascular imaging. Iodine maps allow for the direct quantification of contrast medium distribution and improve material differentiation (e.g., hemorrhage vs. contrast enhancement) [22,23,24,25], whereas the use of VNC imaging has shown a great benefit in radiation dose reduction, substituting true unenhanced CT phases in selected cases [26,27,28].

Previous studies have evaluated DECT VMI+ for vascular assessment [29,30]. Wichmann et al. [31] evaluated the image quality and diagnostic accuracy of low-keV VMI+ compared to the standard VMI algorithm and the conventional series (F_0.5), and they concluded that 40–50 keV are the most recommended VMI+ reconstructions for lower extremity runoff. Furthermore, Leithner et al. [32] demonstrated increased suitability of 40-keV VMI+ images for carotid and intracranial artery evaluation, with an excellent assessment of stenosis. Moreover, low-keV VMI+ has been shown to reduce the contrast medium dose needed to obtain good image quality with high diagnostic value [33,34]. 

Our results support the high image quality and diagnostic suitability of low-energy VMI+ images in the evaluation of lower-extremity arteries in patients with DM. 

In this context, DM increases the risk of PAD and accelerates its progression, leading to a high probability of significant stenotic lesions that impair the iodine signal in distal vessels. 

The main pathogenic processes of PAD in diabetic patients are correlated to an increased state of vascular inflammation, endothelial dysfunction, vasoconstriction, platelet activation, and thrombotic risk [35].

Furthermore, DM-induced CKD represents a strong unique risk factor, promoting PAD risk and severity [6,7,36]. The underlying mechanisms linking the two diseases are not fully understood, but inflammation, oxidative stress, and endothelial dysfunction play a crucial role [37]. In this context, several studies have shown a worse prognosis of PAD in patients with CKD compared to those without [38,39,40]. 

Thus, the reduction in contrast medium dose in patients with DM may represent a huge benefit, preserving residual renal function from the contrast medium nephrotoxicity and the risk of contrast agent-induced nephropathy [41,42,43]. 

This is particularly relevant in patients with PAD and frequently existing comorbidities, who undergo CTA for diagnosis, pre-operative planning, and subsequent endovascular treatments, which often require large quantities of contrast medium due to their complexity. 

Therefore, the above-mentioned advantages of using VMI+ may represent crucial points with effects on the health, economy, and resource consumption.

This study has limitations that have to be addressed. First, all CT scans have been performed on a single DECT scanner with dual-source technology. Results from other DECT technology may be different and must be further evaluated. Second, the ROI drawing was difficult in some cases due to vessel disease (calcifications and plaques) and may have affected the analyses. Third, the retrospective study design may have limited the generalizability of our results; further studies are necessary to confirm these findings as well as to determine their clinical benefit. Fourth, we arbitrarily chose energy levels from 40 to 100 keV, with an increment of 15 keV; results may be different at unrated energy levels.

In conclusion, DECT 40-keV VMI+ showed the highest values of SNR and CNR in quantitative evaluation, followed by the 55-keV VMI+. Regarding subjective vessel assessment, 55-keV VMI+ received the best scores.

Thus, the use of low energy level VMI+ reconstructions (40 to 55-keV) should be deployed in clinical practice in order to improve contrast and image quality when analysing CTA examinations of lower extremities in patients with DM and PAD. Especially for diabetic patients, who are usually affected by CKD and have an increased risk of contrast-induced nephropathy, reduction in contrast media dose used during CTA could be an additional benefit of low-keV VMI+.

## Figures and Tables

**Table 1:** Five-point Likert Scale for subjective analysis of image quality, image noise, and vessel contrast.

	Image Quality	Image Noise	Vessel Contrast
1	Very poor	Major noise	Not diagnostic
2	Poor	More than average noise	Poor
3	Acceptable	Average noise	Acceptable
4	Good	Minor noise	Good
5	Optimal	None	Optimal

**Table 2:** Patient Demographics.

Patient Demographics	
Characteristic	All Patients (*n* = 77)
Sex, no. (%)	
Male	41 (53%)
Female	36 (47%)
Mean age; SD (y)	79.17 ± 6.46
Male	78.93 ± 6.05
Female	79.44 ± 6.99
Diagnosed Comorbidities	
Peripheral arterial disease	34 (44%)
Hypertension	43 (56%)
Kidney Insufficiency	16 (20%)
Coronary artery disease	23 (30%)
Previous myocardial infarction	9 (12%)

**Table 3:** Results of the quantitative measurements in the anterior tibial artery (ATA), fibular artery (FA), and posterior tibial artery (PTA).

Parameters	40 keV	55 keV	70 keV	85 keV	100 keV	F_0.5
Attenuations						
ATA	1174.97 ± 284.23	654.73 ± 145.84	407.39 ± 84.02	281.43 ± 53.06	210.85 ± 36.14	249.43 ± 29.08
FA	1138.28 ± 319.33	636.71 ± 171.39	391.74 ± 102.12	273.76 ± 64.78	205.94 ± 46.09	259.21 ± 31.21
PTA	1227.97 ± 254.92	681.01 ± 136.60	419.15 ± 80.77	285.62 ± 52.81	209.79 ± 35.89	245.33 ± 29.95
Average all vessels	1180.41 ± 45.09	657.48 ± 22.28	406.09 ± 13.75	280.27 ± 6.01	208.86 ± 2.58	251.32 ± 7.13
Noise	40.34 ± 5.72	28.97 ± 5.57	21.36 ± 4.60	19.64 ± 3.25	19.92 ± 2.99	20.75 ± 5.48
SNR						
ATA	29.84 ± 8.32	23.22 ± 6.57	20.02 ± 5.96	14.52 ± 3.60	11.01 ± 2.42	13.25 ± 4.34
FA	28.95 ± 9.35	22.55 ± 7.21	19.27 ± 6.45	14.14 ± 4.16	10.75 ± 2.95	13.64 ± 4.38
PTA	30.94 ± 8.04	24.06 ± 6.27	20.56 ± 5.87	14.82 ± 3.53	10.96 ± 2.55	12.77 ± 4.33
Average all vessels	29.91 ± 0.99	23.28 ± 0.76	19.95 ± 0.65	14.49 ± 0.34	10.91 ± 0.14	13.22 ± 0.44
CNR						
ATA	28.55 ± 8.22	21.42 ± 6.31	17.59 ± 5.54	12.15 ± 3.35	8.54 ± 2.14	10.54 ± 3.36
FA	27.60 ± 9.24	20.71 ± 7.00	16.83 ± 6.20	11.76 ± 4.00	8.28 ± 2.72	10.98 ± 3.45
PTA	29.65 ± 7.90	22.26 ± 6.02	18.13 ± 5.52	12.41 ± 3.34	8.46 ± 2.27	10.20 ± 3.57
Average all vessels	28.60 ± 1.03	21.46 ± 0.78	17.52 ± 0.66	12.10 ± 0.33	8.42 ± 0.13	10.57 ± 0.39

All data are displayed in mean ± SD. SNR: signal-to-noise ratio; CNR: contrast-to-noise ratio.

## Data Availability

The data presented in this study are available on request from the corresponding author. The data are not publicly available due to privacy and ethical reasons.

## Abbreviations

ATA	Anterior Tibial Artery
CKD	Chronic Kidney Disease
CNR	Contrast-to-Noise Ratio
CTA	CT Angiography
DECT	Dual Energy CT
DM	Diabetes Mellitus
FA	Fibular Artery
PAD	Peripheral Arterial Disease
PTA	Posterior Tibial Artery
ROI	Region of Interest
SD	Standard Deviation
SNR	Signal-to-Noise Ratio
VMI	Virtual Monoenergetic Image
